# Aromatherapeutic and Antibacterial Properties of Cotton Materials Treated with Emulsions Containing Peppermint Essential Oil (*Menthae piperitae aetheroleum*)

**DOI:** 10.3390/polym15102348

**Published:** 2023-05-17

**Authors:** Genoveva Rosu, Emil Ioan Muresan, Adrian Florin Spac, Mariana Diaconu, Diana Elena Ciolacu, Angela Danila, Carmen Tita, Augustin Muresan

**Affiliations:** 1 Faculty of Industrial Design and Business Management, Gheorghe Asachi Technical University of Iasi, 29 Prof. Dr. Docent D. Mangeron Blvd, 700050 Iasi, Romania; 2 Organic, Biochemical and Food Engineering Department, ‘Cristofor Simionescu’ Faculty of Chemical Engineering and Environmental Protection, “Gheorghe Asachi” Technical University of Iasi, 73 Prof. Dr. Docent D. Mangeron Blvd, 700050 Iasi, Romania; 3 Department of Phisico-Chemistry, Faculty of Pharmacy, “Grigore T. Popa” University of Medicine and Pharmacy, 16 Universității Street, 700115 Iași, Romania; 4 Department of Environmental Engineering and Management, ‘Cristofor Simionescu’ Faculty of Chemical Engineering and Environmental Protection, “Gheorghe Asachi” Technical University of Iasi, 73 Prof. Dr. Docent D. Mangeron Blvd, 700050 Iasi, Romania; 5 Department of Natural Polymers, Bioactive, and Biocompatible Materials, “Petru Poni” Institute of Macromolecular Chemistry, 41A Grigore Ghica Voda Alley, 700487 Iasi, Romania

**Keywords:** antibacterial, aromatherapeutic, beeswax, cellulose, chitosan, emulsion, essential oil, gelatin, Tween 80

## Abstract

The objective of the work was to obtain materials with aromatherapeutic and antibacterial properties by applying emulsions based on peppermint essential oil (PEO) onto cotton fabric. For this purpose, some emulsions based on PEO incorporated in various matrices (chitosan + gelatin + beeswax; chitosan + beeswax; gelatin + beeswax and gelatin + chitosan) were prepared. Tween 80 was used as a synthetic emulsifier. The influence of the nature of matrices and of the concentration of Tween 80 on the stability of the emulsions was evaluated by the creaming indices. The materials treated with the stable emulsions were analyzed in terms of sensory activity, of the comfort characteristics, and of the gradual release of the PEO in the artificial perspiration solution. The sum of volatile components retained by samples after exposure to air was determined by GC-MS. The results regarding antibacterial activity showed that materials treated with emulsions have a good inhibitory effect on *S. aureus* (diameters of the inhibition zones between 53.6 and 64.0 mm) and on *E. coli* (diameters of the inhibition zones between 38.3 and 64.0 mm). Our data suggest that by applying peppermint-oil-based emulsions on a cotton support, aromatherapeutic patches, bandages and dressings with antibacterial properties can be obtained.

## 1. Introduction

In recent years, essential oils have attracted both the attention of researchers as well as of consumers due to their aromatherapeutic [1,2], antibacterial [3,4,5,6,7], anti-inflammatory [5,7] and antioxidant [5,6,7] properties, representing an alternative to the use of antibiotics or other chemical additives. The rapid development of microorganisms’ resistance to drugs is becoming a worldwide problem. Essential oils (EOs) that have a complex chemical composition, containing various chemical compounds, such as terpenes, phenylpropanoids, aldehydes, ketones, alcohols, esters, phenols, ethers, and others, can be considered an innovative alternative to replace drugs due to their biological activity against a series of pathogenic microorganisms, such as bacteria, viruses, and fungi [8,9]. Used in aromatherapy, essential oils could represent a standalone therapeutic method or a complementary therapy to conventional medicine contributing to the improvement in the physical, emotional and spiritual states of healthy people, as well as patients with various diseases [10,11,12,13,14,15]. For instance, aromatherapy with peppermint and lavender essential oils can reduce fatigue in cardiac patients [10].

Essential oils are complex mixtures of volatile compounds extracted from different parts of plants (such as flowers, leaves, stems, roots, fruits, and bark), by various methods (i.e., by steam distillation, cold pressing, solvent extraction and so on) [16]. The loss of biologically active principles through evaporation, high sensitivity to environmental factors, and the difficulties related to the controlled release limit the commercial application of essential oils [17]. For this reason, it is necessary to formulate essential oils in different forms: emulsions, microcapsules and gels. The incorporation of essential oils into various biopolymers such as gums, modified starch, gelatin, sodium alginate, chitosan in wax or in β-ciclodextrine, allows the protection and release of the essential oils over time [18,19,20,21,22,23,24,25,26,27,28,29].

Various studies approaching the modification of the surfaces of textile supports in order to obtain dressings with antibacterial and anti-inflammatory activities with a wide spectrum of action have been reported, for example, by applying nanoparticles containing silver and zinc on the surface of cotton fabric [30], by the functionalization of a cotton gauze with gallic acid [31], and by coating with natural polymers [32]. There have been studies in which cotton materials were impregnated with various emulsions containing essential oils to increase release time, therapeutic effectiveness, comfort, etc. Through the application of essential oils on textiles a controlled time release of the biologically active ingredients can be achieved. For example, for this purpose, essential oils from rosemary [33], thyme [34], rose and sage [35], and peppermint were used [36] among others.

The introduction of essential oils used as active substances within polymeric matrices based on polyvinyl alcohol/polyvinyl pyrrolidone (PVA/PVP) offers an effective opportunity for their use in various wound dressings [37].

Emulsions used as essential oil release systems have earned particular interest due to their properties (ease of preparation, stability over time, translucent appearance) [38,39,40,41]. Due to its properties, peppermint essential oil is one of the most popular oils, being widely used in medicine and the food industry. The choice of peppermint essential oil was based on the fact that it has a pleasant smell and a cooling effect, but also has some important biological activities. Thus, peppermint essential oil, consisting mainly of menthol, menthone, menthene, β-caryophyllene, camphene and other compounds, is a mixture of biologically active secondary metabolites with anti-inflammatory, immunomodulatory, antibacterial, antiviral, scolicidal, antioxidant, antitumor, antifatigue, neuroprotective, and other activities [7,42].

Peppermint essential oil is also an effective natural pain reliever, an anti-inflammatory and a good antimicrobial and antioxidant agent due to its monoterpene and ketone content [43,44,45,46,47,48,49,50,51]. Due to its fragrance and therapeutic properties, peppermint essential oil is used in aromatherapy to exhibit relaxing and toning effects on tissues [52,53,54,55]. Peppermint essential oils were found to inhibit the growth of Gram-positive and Gram-negative bacterial strains, the obtained results being comparable to those of the antibiotic gentamicin. The essential oils showed a wider spectrum of activity but lower inhibiting activity compared to the investigated commercial antibiotic. The biological activity, pharmacological effects, and reduced toxicity make peppermint essential oil efficient in treating injuries caused by burns, wounds, and in treating psoriasis [51].

Considering the antimicrobial and antifungal properties of peppermint essential oil, it can be used in the manufacturing of antibacterial dressings and food packaging, and for improving indoor air quality.

Materials with aromatherapeutic properties are obtained by the treatment of cotton fabrics with emulsions or hydrogels containing various essential oils of rosemary [33,56], thyme [34], geranium [57], citrus [58], peppermint [59] and others. The essential oils are incorporated into polyester and cotton fabrics by different methods, such as ultrasound, diffusion, padding [57] or by immobilization onto textile materials of the microcapsules containing essential oils (using methods such as bath exhaustion, padding, dry curing pad, chemical grafting, printing, coating, spraying, or immersion) [58]. Encapsulation can be performed at the micro and nanoscale level, with polymeric materials usually being used to incorporate the essential oils [59,60].

This work aims to obtain textile materials with aromatizing and antibacterial proper-ties by applying emulsions containing essential peppermint oil. The prepared emulsions differ from each other by their concentration of Tween 80 and by the nature of the essential oil incorporation agent. The novelty of the work consists of reducing the amount of synthetic emulsifier Tween 80 by using natural, ecological biodegradable polymers as co-emulsifiers. In addition, wax and gelatin used as incorporation agents of MEO are cheap products that are easy to procure. The release kinetics of the essential oils from the textile materials were analyzed according to the Korsmeyer—Peppas model.

## 2. Materials and Methods

### 2.1. Materials

Peppermint essential oil (MEO) was purchased from S.C. Fares S.A, Orăștie, Romania, Tween 80 was supplied by Merck (Darmstadt, Germany), the vegetable glycerin (purity 99.5%) was supplied by S.C. Comecom SRL Bucharest (Bucharest, Romania), the beeswax was procured from a private apiary in the northern-eastern region of Romania, and the gelatin was purchased from S.C. Lesaffre Romania SRL. The chitosan, with molecular weight 100,000–300,000 g∙mol^−1^ was supplied by Fluka Chemie GmbH, Switzerland. The chitosan solution was obtained by the complete dissolving of chitosan into a 1% acetic acid solution. n-Hexane with 96% purity was purchased from Merck, SUA.

### 2.2. Preparation of Emulsions

The emulsions were prepared as follows: (i) the beeswax was melted at 75 °C; (ii) the solutions containing distilled water, Tween 80, gelatin, glycerin, and chitosan (prepared according to the recipes presented in Table 1) were warmed up under stirring at 70 °C. Solution (ii) was added gradually over the melted beeswax and the stirring continued at 70 °C for 30 min. The obtained emulsion was cooled to 60 °C and peppermint essential oil was added to it dropwise. Thereafter, stirring was continued for 30 min using a high-speed homogenizer (T25 Ultra-Turrax^®^, IKA, Taufkirchen, Germany).

### 2.3. Application of the Emulsions onto Textile Support

The experiments were conducted on textile supports made of 100% cotton fabric, washed and bleached having a mass of 160 g/m^2^. Cellulosic materials were used in the experiments since these materials have a high liquid absorption capacity and an antistatic behavior. The cotton fabric samples measuring 10 cm × 10 cm (1.6 g) were impregnated with the freshly prepared emulsions using a Benz (Zurich, Switzerland) padding foulard at a squeezing degree of 160% (1 g of material corresponds to 1.6 g of emulsion). After padding, each sample was (i) immediately introduced into a sealed tight plastic bag for the release study of the essential oil into an artificial perspiration solution or (ii) exposed to air in order to study the release of the volatile components.

### 2.4. Characterization of Emulsions

#### 2.4.1. Microscopic Evaluation of the Emulsions

Microscopic evaluation was carried out using the KRUSS optical microscope, and the microscopic images were captured for computerized analysis using a photo-digital camera (Nikon, Coolpix P 5100).

#### 2.4.2. pH Measurements

The pH values of the emulsions were measured with a WTW InoLab pH 720 pH meter (Nitech, Chișinău, Republic of Moldova).

#### 2.4.3. Creaming Index (CI)

For stability tests, the emulsion samples were poured into 10 mL graduated glass cylinders and stored at ambient temperature. The creaming index (*CI%*) was calculated according to Equation (1) [61]:

(1) $$CI\%=100\cdot \frac{H_{C}}{H_{0}}$$

where *H*_0_ is the total height of the emulsion layer and $H_{C}$ is the height of the cream layer. Analyses were performed in triplicate. The heights $H_{C}$ and *H*_0_ were measured directly using the graduated scale of the glass cylinder (after 4, 24, 48, and 72 h).

### 2.5. Characterization of the Materials Treated with the Obtained Emulsions

#### 2.5.1. Statistical Analysis

In order to analyze the differences between the properties of the obtained samples, the statistical method was used using a one-way variation (ANOVA) with a significance level of *p* < 0.5. The obtained data were processed on the computer using the Origin 6.1 program (OriginLab, Northampron, MA, USA).

#### 2.5.2. Sensorial Evaluation

The samples treated with the peppermint essential oil emulsions were kept at room temperature and smelled after every five days. The sensorial evaluation was performed by a group of 10 subjects.

#### 2.5.3. SEM-EDX Analysis

The morphology of the textile materials was evaluated by using a Scanning Electron Microscope (SEM) type Quanta 200 (FEI Company, Hillsboro, OR, USA), operating at an accelerating voltage of 15 kV, with secondary electrons in low vacuum mode. The microscope was coupled with an Energy Dispersive X-ray (EDX) system to perform the elemental analysis.

#### 2.5.4. Attenuated Total Reflectance—Fourier Transform Infrared Spectroscopy (ATR-FTIR) Analysis

ATR–FTIR measurements were performed on a Bruker Vertex 70 FTIR spectrometer equipped with a Bruker Optics variable angle accessory. Spectra were recorded from 4000 to 600 cm^−1^, at a resolution of 4 cm^−1^.

#### 2.5.5. GC-MS Method

The chemical analyses of the hexane extracts of peppermint essential oil were determined by GC-MS. A gas chromatograph Agilent Technologies 7890 A type, equipped with an Agilent Technologies 7683B Series Injector autosampler and an Agilent Technologies 5975C inert mass spectrometer MSD with Triple Axis detector (all from Agilent Technologies, Santa Clara, CA, USA) was used with a DB-5MS capillary column (30 m × 0.25 mm i.d., 0.25 μm film thickness). The flow rate of the helium carrier gas was set at 1.0 mL/min, the split ratio was set at 1:100 and a sample volume of 0.25 μL was injected. The injector temperature was 250 °C. In the oven, the initial temperature of 40 °C was kept constant for 3 min, then it was increased by 10 °C/min up to 280 °C, remaining constant for another 3 min. For the MS detector, the temperatures of the MS Source was 230 °C and of MS Quad was 150 °C. The detection parameters: Solvent delay—3 min, mass range—15 to 300 *m*/*z*, detection mode—SCAN. The software used was Agilent Technologies MSD ChemStation E 02.00.493.

Identification of the volatile compounds found in the samples extracted in hexane was performed using the Wiley Mass Spectra library. By integrating the obtained chromatograms, the area of the peaks of the volatile components from the extracts in hexane was determined and their sum was calculated. Considering as a starting point the sample extracted in hexane at a time of 0 h after impregnation (that is, a content of 100%), the percentage was then calculated for the samples that were in contact with air for 3 and 6 h, respectively.

#### 2.5.6. Release Study of the Peppermint Essential Oil

Taking into account the possible uses of materials treated with emulsions containing peppermint essential oil (aromatherapeutic patches, bandages and wound dressings), the release of the volatile components was studied in two ways:

- release in artificial perspiration solutions;
- the release of volatile components into the air.

##### Release of Essential Oil in Artificial Perspiration Solutions

To determine the quantity of essential oil released over time, a known amount of cotton material treated with emulsion was finely sliced, introduced into 50 mL of artificial perspiration solution, and maintained under constant stirring (60 rpm) at 37 °C. At pre-established time intervals, the solution was analyzed using the Camspec M501 Single Beam Scanning UV/Visible spectrophotometer by measuring the absorbance at a wavelength of 275 nm using the artificial perspiration solution as standard.

The peppermint essential oil release mechanism was evaluated using the Korsmeyer—Peppas Equation (2) [62]:

(2) $$\frac{C_{t}}{C_{\infty }}=k_{\mathrm{KP}}\cdot t^{n},$$

where C_t_ is the amount of peppermint essential oil released in time t, $C_{\infty }\;$ is the amount of oil released at equilibrium, $k_{\mathrm{KP}}$ is the rate constant, and n is the diffusion exponent used to characterize different release mechanisms.

##### Release of Volatile Components into the Air

To determine the composition of volatile substances in the peppermint essential oil, a 2% solution of essential oil in hexane was prepared. To determine the residual content of peppermint essential oil on the cotton fabric, three cotton fabric samples (10 × 10 cm; 1.6 g) were impregnated with a freshly prepared emulsion (E1, E3 and E6). The samples were exposed to air for 0 h (samples marked TE1.0, TE3.0 and TE6.0, respectively), 3 h (samples marked TE1.3, TE3.3 and TE6.3, respectively) and 6 h (samples marked TE1.6, TE3.6 and TE6.6, respectively). The peppermint essential oil from the impregnated fabric samples was extracted in a volume of 8 mL of hexane by placing them in a hermetically sealed vessel for 6 h, with periodic shaking once every 30 min. The hexane solution obtained was analyzed by gas chromatography with detection by mass spectrometry, under the mentioned conditions.

#### 2.5.7. ‘In Vitro’ Evaluation of the Antibacterial Activity

The tests were performed using two bacterial species: *Escherichia coli* (Gram-negative bacteria) and *Staphylococcus aureus* (Gram-positive bacteria), both isolated from contaminated water samples (from the collection of microbiology laboratory, ‘Cristofor Simionescu’ Faculty of Chemical Engineering and Environmental Protection, Iasi, Romania). The antimicrobial activity was tested in vitro under the optimal and standardized bacteria growing conditions (growing medium: YPG extract of peptone-glucose-agar, inoculum, incubation time, temperature) in the presence of the cotton fabric samples (circular shape, 20 mm in diameter) by using the diffusion method [63].

This method is based on the property of the chemical species to diffuse in the culture media distributed in sterile Petri plates at a certain distance, achieving a concentration gradient that decreases proportionally with the distance.

The preparations were applied on the surface of the culture media, after it was seeded with a cellular suspension of tested microorganisms 5 × 10^7^ CFU/mL and incubated at 37 °C for 24–48 h in a thermostat (Ecocell) for microorganism growth. The results were determined by measuring the diameter of the inhibition zone in mm (2 to 3 times in different directions). The influence of the treated cotton fabrics toward tested microorganisms was expressed as the inhibition degree of living cellular growth (I[%]) and is calculated as in the equation:

(3) $$I\left(\%\right)=\left[\left(A-B\right)\cdot 100\right]/A$$

where A is the diameter of the cellular growth zone in control (mm) and B is the diameter of the cellular growth zone in the sample (mm).

#### 2.5.8. Analysis of the Comfort Indices

The comfort indices for the studied samples were determined as follows: hygroscopicity was determined according to the Standard EN ISO 12571:2000, permeability of water vapors was determined according to ISO 11092:1993 on a Permetest (Sensora, Czech Republic) apparatus, and the air permeability was determined according to SR EN ISI 9237 on the METEFEM (Budapest, Hungary) apparatus, using a pressure difference of a 10 mm water column.

## 3. Results and Discussions

### 3.1. Optical Microscopy

**Figure 1:**
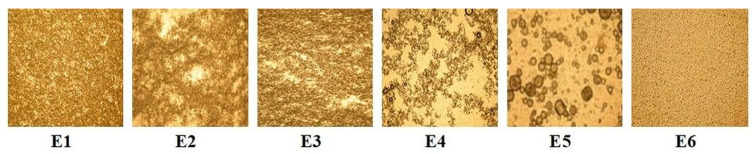
Optical microscopic images for the emulsions containing peppermint essential oil.

The stability of the emulsions is an important factor for their evaluation. Figure 1 presents the microscopic images of all emulsions after 1 h from their preparation.

From the presented microphotographs, we can notice that the emulsions E1, E3, and E6 show a texture with a greater number of small particles and a more uniform distribution of the particle size, which provides them with time stability. All images were taken at the same degree of magnitude (×10). Samples E1, E3 and E6 (which showed good stability) were further studied. Samples E2, E4 and E5 were not studied because they did not show good stability over time (probably because during measurement of samples E2 and E4 the phenomenon of flocculation was occurred, while at that of sample E5 the coalescence phenomenon occurred).

### 3.2. pH Determination

The pH of the emulsion is an important parameter considering the destination of the treated fabrics (as health products). pH values for the treated samples are shown in Table 2.

The skin pH is normally acidic with pH values in the range of 4–6. Since the obtained emulsions have pH values between 5.3–5.7, it can be concluded that all these materials are suitable for topical applications.

### 3.3. Stability of the Prepared Emulsions

**Figure 2:**
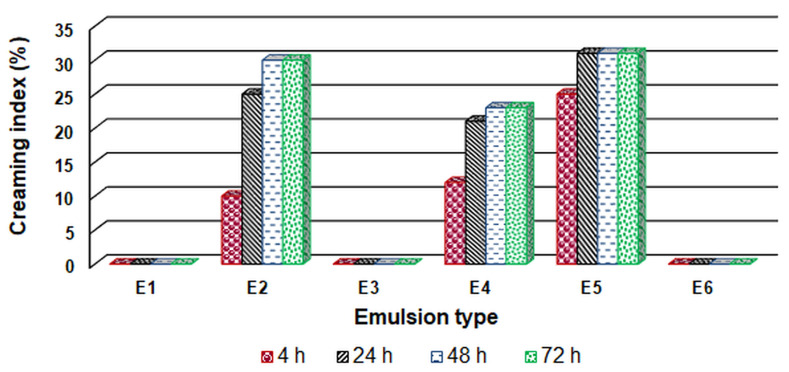
Variation in time of the creaming index values for the emulsions containing essential peppermint oil.

The emulsion stability was evaluated by the creaming index. The calculated values of the creaming index are shown in Figure 2.

According to the obtained results, the best stability was exhibited by emulsions E1, E3, and E6, while emulsion E2 (which contained the least amount of emulsifier Tween 80), emulsion E4 (that did not contain gelatin) and emulsion E5 (that did not contain chitosan) exhibited lower stability. The synthetic emulsifier Tween 80 has the role of ensuring a fine dispersion of the oil particles by reducing the surface tension at the oil/water interface. Decreasing the concentration of Tween 80 (less than 1.5%) decreases the surface tension which is the thermodynamically driving force responsible for the coalescence of oil droplets. Chitosan and gelatin can be considered as amphiphilic macromolecules that play an essential role in the stabilization of emulsions. By forming complex structures between gelatin and chitosan (through van der Waals interactions and hydrogen bonds), improved functional properties resulted compared to gelatin and chitosan used alone. This compact network of chitosan-gelatin formed around the oil droplets created protective films that hindered the collision of the droplets, preventing the phenomena of coalescence and flocculation [64,65].

### 3.4. Characterization of the Emulsion-Treated Cotton Samples

Considering that emulsions E1, E3, and E6 were the most stable over time, the samples treated with these emulsions coded as TE1, TE3 and TE6 were the ones used in the further investigations. The untreated sample was denoted as UT.

#### 3.4.1. Sensorial Evaluation

Textile materials are excellent media for transferring fragrance compounds. The samples treated with the prepared emulsions were kept at room temperature and were smelled every day. The sensorial evaluation was performed by a group of 10 subjects. Table 3 presents the average values obtained from the sensorial evaluation.

From the obtained data it can be concluded that the intensity of the smell decreases over time. The wax-containing samples (TE1 and TE3) retain the odor intensity for a longer period compared to the wax-free TE6 sample.

#### 3.4.2. SEM-EDX Analysis

**Figure 3:**
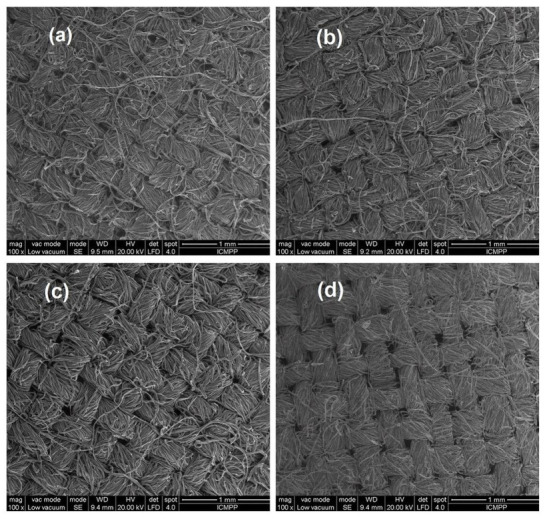
Scanning electron microscope images: (**a**) sample TE1; (**b**) sample TE3; (**c**) sample TE6 and (**d**) the untreated cotton sample UT.

SEM images for the untreated cotton sample and the treated cotton samples are presented in Figure 3.

From the analysis of the SEM microphotographs, it can be noticed that the application of the emulsion onto the surface of the textile support caused a reduction in the spaces between the cotton fibers. This modification of the surface explains the lower air permeability of the samples treated with the emulsion, which varied between 12.72 m^3^/m^2^∙min and 14.11 m^3^/m^2^∙min compared to the air permeability of the untreated cotton sample which had a value of 37.16 m^3^/m^2^∙min.

**Figure 4:**
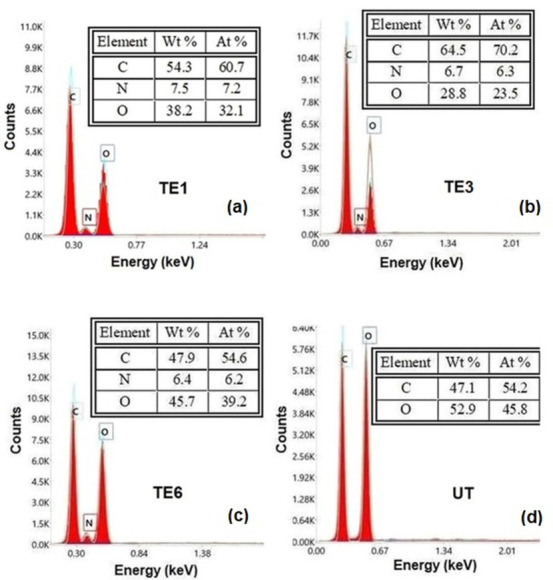
EDX elemental analysis of the cotton samples.

In the case of the EDX analyses, for each sample three determinations were carried out (within each set of determinations, the differences between the calculated mean value and the values corresponding to the three determinations were not greater than ±1%). Figure 4 shows the EDX spectra of the investigated samples (for each set of analyses, the spectrum was selected whose experimental values were the closest to the average values calculated).

EDAX analyses of the samples TE1, TE3, and TE6 confirmed the presence of nitrogen coming from chitosan and gelatin, respectively, contained by the E1, E3, and E6 emulsions applied to the textile support: Figure 4a (sample TE1), Figure 4b (sample TE3), Figure 4c (sample TE6). For the sample TE6 treated with the E6 emulsion, which did not contain beeswax, a change in the C/O ratio was observed (Figure 4d (sample UT)). EDX analysis of the untreated cotton sample UT indicated only the presence of C and O elements (Figure 4d (sample UT)).

#### 3.4.3. FTIR Analysis

**Figure 5:**
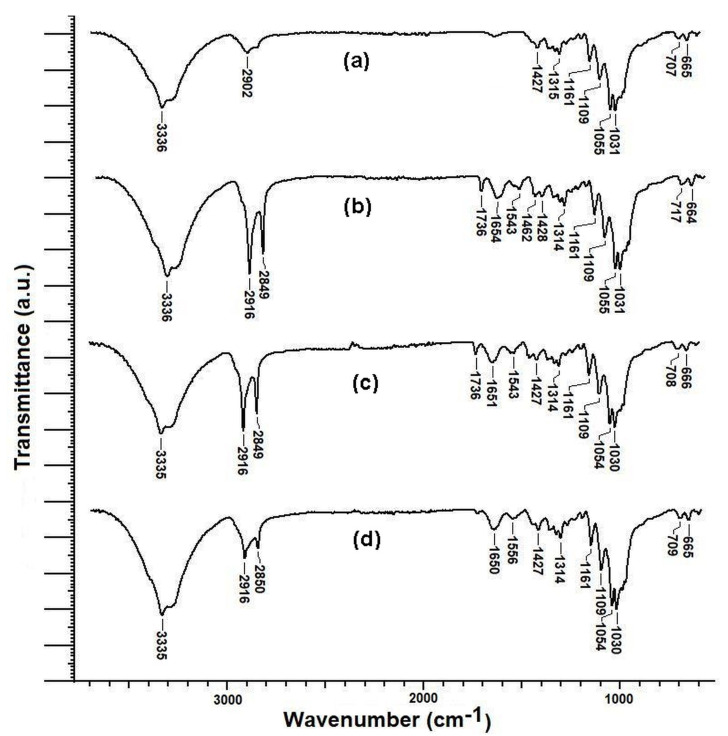
FTIR spectra of the (**a**) untreated sample UT; (**b**) sample TE1; (**c**) sample TE3 and (**d**) sample TE6.

The Fourier transform infrared (FTIR) spectra of the untreated cotton sample and the cotton samples treated with the emulsions E1, E3, and E6 are shown in Figure 5.

The strong and wide absorption band recorded at 3336 cm^−1^ corresponds both to the stretching vibrations of the O-H bonds within the hydroxyl groups of cellulose for the untreated sample (Figure 5a), and to the stretching vibrations of the N-H bonds within the amino groups of the chitosan and gelatin contained by the emulsions applied on the cotton samples (Figure 5b–d). In the case of the untreated sample, the absorption bands located at 2916 cm^−1^ and 2849 cm^−1^ are attributed both to the asymmetric stretching vibrations as well as to the symmetrical stretching vibrations of the C-H bonds within the CH_2_ groups of cellulose (Figure 5a). In the case of the samples treated with emulsions, the intensity of these two peaks increased due to the CH_2_ groups present in chitosan, gelatin, and beeswax (Figure 5b–d). The peak at 1736 cm^−1^, which appears in the case of the samples treated with beeswax-containing emulsions, is attributed to the C=O bonds from the ester groups of the esters contained by beeswax (Figure 5b,c). The peaks from 1651 cm^−1^ and 1543 cm^−1^ can be attributed to the stretching vibrations of the C=O bonds and, respectively, to the bending vibrations of the N-H bond belonging to the amide groups from gelatin (Figure 5b–d). The peaks located at 1427 cm^−1^ and 1315 cm^−1^ correspond to the in-plane bending (rocking) vibrations of C-H bonds from the CH_2_ groups of cellulose, chitosan, beeswax, and from the CH_3_ groups of gelatin and beeswax. The peaks in the range 1161 cm^−1^–1031 cm^−1^ are attributed to the stretching vibrations of the C–O bonds within the C–O–C glycoside linkages present in cellulose and chitosan. The peaks located around 700 cm^−1^ are attributed to the out-of-plane bending (twisting and wagging) vibrations of the C-H bonds from the CH_2_ groups.

#### 3.4.4. Release of Essential Oil in Artificial Perspiration Solution

**Figure 6:**
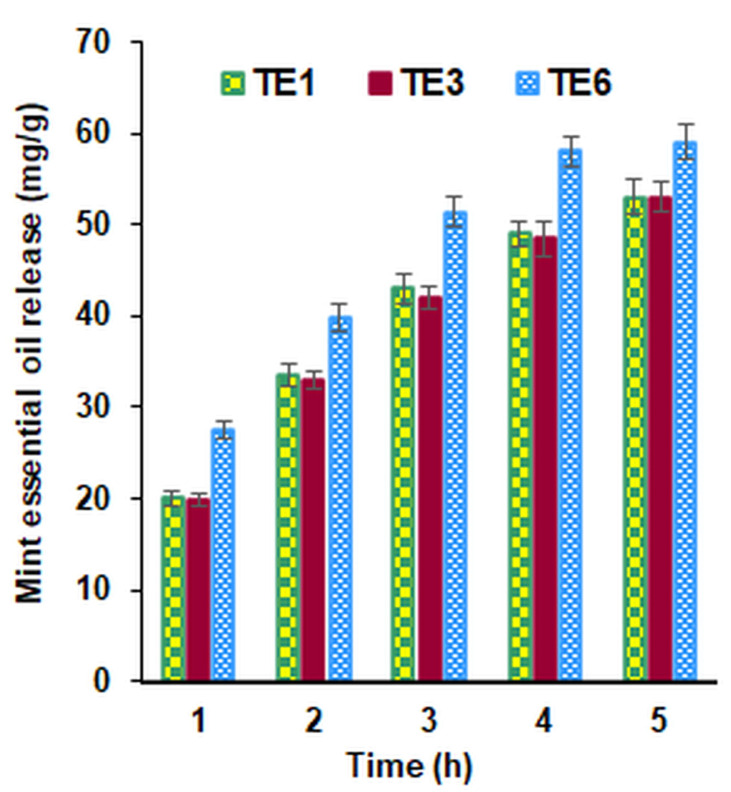
The profile for time release of the biologically active compound from the treated samples (TE1, TE3 and TE6).

Essential oils are gradually adsorbed by the skin from the textile fabric by a release mechanism over time. The quantities of peppermint essential oil, which were released from the textile support after 1, 2, 3, 4, and 5 h, respectively, were calculated using the calibration curve of the peppermint essential oil solution y = 1.609·x, wherein x represents the concentration of the biologically active compounds, and y is the absorbance at 275 nm. The controlled release of the peppermint essential oil from the emulsion-treated materials allowed the biologically active compounds to act gradually over a longer period (Figure 6).

Analyzing the obtained results, it was observed that in the case of the samples treated with the E6 emulsion, which did not contain beeswax, the essential oil was released in a shorter period. The same amount of oil was released more slowly, for a longer time, for the samples treated with the emulsions E1 and E3 that contain beeswax, gelatin, and chitosan. The slower release in time of the peppermint essential oil from these samples could be explained on the one hand by the greater affinity that the hydrophobic beeswax exhibits towards the oil particles and on the other hand by the fact that the chitosan and gelatin-containing emulsions reduce the spaces between the textile fibers, which causes a more difficult release of the essential oil.

**Figure 7:**
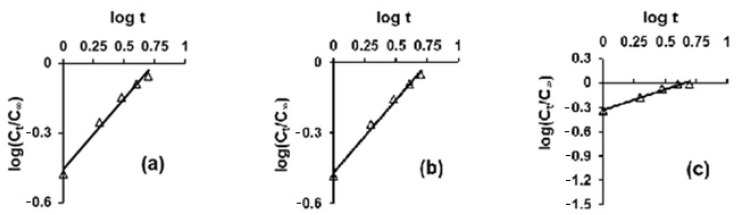
Korsmeyer–Peppas kinetic model for the release of the MEO from the samples treated with the emulsions: (**a**) TE1, (**b**) TE3, (**c**) TE6.

The Korsmeyer–Peppas model was used to elucidate the release kinetics of the peppermint essential oil from the textile materials treated with the prepared emulsions. The experimental data analyzed according to this model are shown in Figure 7.

The kinetic parameters corresponding to this kinetic model are shown in Table 4. The values of R^2^ which are higher than 0.962 indicate that the essential oil was released according to the Korsmeyer—Peppas kinetic model.

The “n” is a constant which characterizes the rate of release. The values higher than 0.5 (values ranged between 0.510 and 0.617) suggest that the release of the biologically active compound is controlled from case to case (depending on the nature of the matrix) by diffusion or by swelling followed by diffusion. The highest value of the k_KP_ constant indicates a higher release rate of the essential oil from the cotton sample treated with the emulsion E6 (the sample TE6 which does not contain wax) compared to the samples treated with the other emulsions containing wax (which retains the oil particles more strongly due to its hydrophobic character).

#### 3.4.5. Release of the Volatile Components into the Air

The solution of peppermint essential oil in 2% hexane, and the extracts in the hexane of the cotton fabric samples impregnated with emulsions and exposed to air for 0, 3 and 6 h, respectively, were analyzed by GC-MS in the method conditions. The chromatograms and the identified compounds were presented in the Supplementary Materials. In the 2% peppermint essential oil in hexane solution, a total of 23 compounds were identified, from which the main components were menthol (62.87%), 3-p-menthene (13.16%), menthone (12.75%), camphene (3.15%), β-caryophyllene (2.59%), and carane (1.93%).

**Figure 8:**
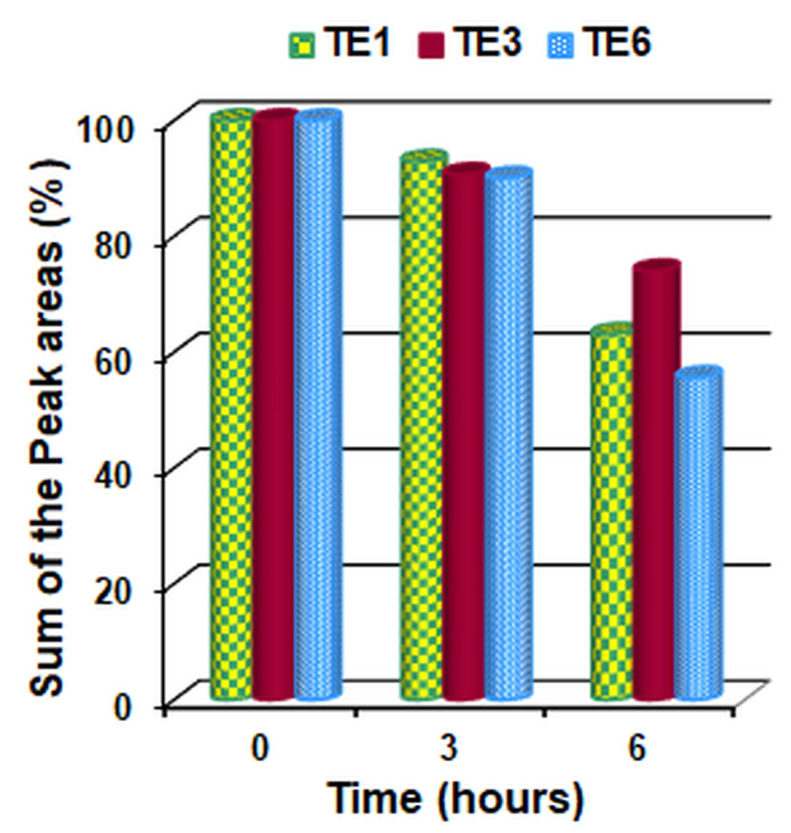
Change over time in the percent of the sum of peak areas of the volatile components from the treated materials (TE1, TE3 and TE6).

**Figure 9:**
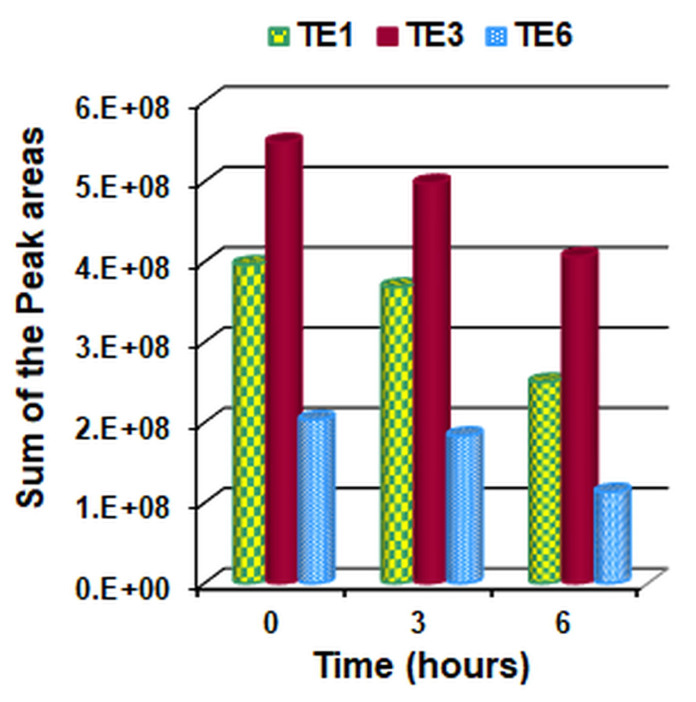
Change in time of the sum of peak areas of the volatile components from the treated materials (TE1, TE3 and TE6).

From the analysis of the chromatograms of the extracts in hexane of the cotton fabric samples it was confirmed that the volatile substances corresponded to those of the peppermint essential oil. The obtained chromatograms and the chemical composition of the peppermint essential oil in hexane and of the extracts in hexane of the cotton fabric samples are presented in the Supplementary Materials (Figures S1–S12 and Tables S1–S13). After integrating the chromatograms and determining the peak area corresponding to the volatile components, the sum of their areas was calculated. The value obtained at the initial moment (0 h) was considered as representing 100%, and the remaining values at the subsequent moments (3 and 6 h, respectively) were calculated as a percentage of the initial value. The data obtained are represented in Figure 8 and Figure 9.

Analyzing the data presented in the tables from the Supplementary Materials and those from Figure 8 and Figure 9, the following can be observed:

- After 3 h of air exposure, in all 3 samples analyzed, volatile components of the essential oil of peppermint were found in the range of 90.10–92.72% compared to the initial moment;
- After 6 h of air exposure, the volatile components of the remaining essential oil of peppermint are 63.12% (sample TE1), 74.08% (sample TE3) and 55.65% (sample TE6).
- The highest amount of initial and residual volatile peppermint oil after 3 and 6 h, respectively, was found in sample TE3 and the lowest amount in sample TE6.
- Thus, sample TE3 not only incorporates but also retains the largest amount of essential peppermint oil.
- The smaller amount of mint essential oil retained by sample TE6 (which did not contain wax) compared to samples TE1 and TE3 (which contained beeswax) can be explained by the fact that wax, a hydrophobic compound, retains the essential oil more strongly in the emulsion, respectively, on the surface of the textile material.

#### 3.4.6. Comfort Characteristics of Treated and Untreated Samples

**Figure 10:**
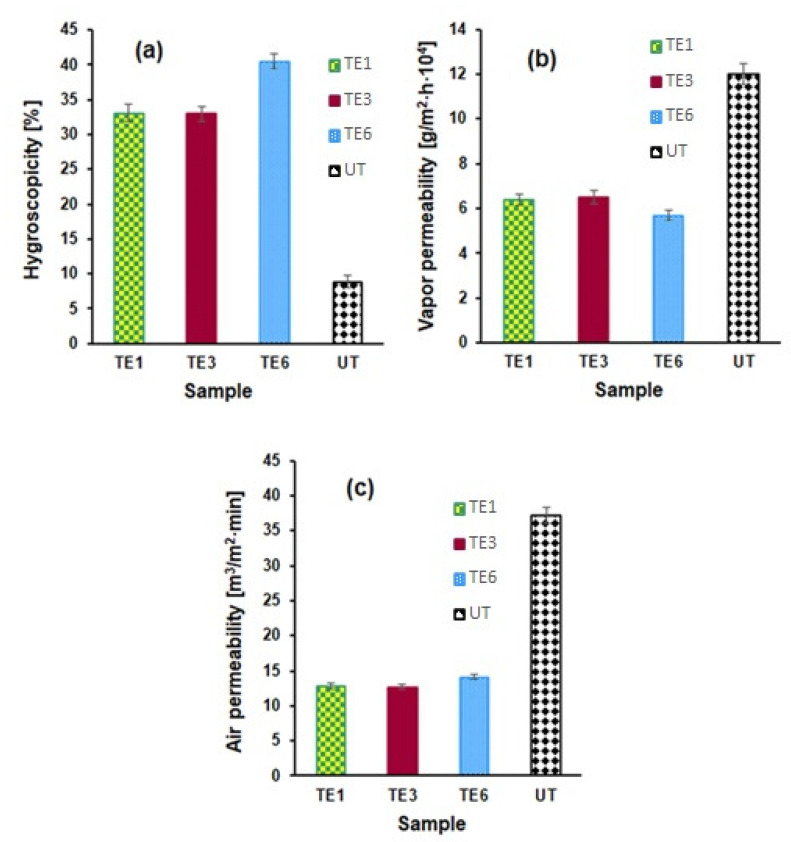
Values of the comfort indexes for the untreated sample (UT) and for the treated samples (TE1, TE3 and TE6): (**a**) hygroscopicity; (**b**) water vapor permeability; and (**c**) air permeability.

Considering the destination of cotton samples treated with peppermint-essential-oil-based emulsions, a significant role is played by the comfort indexes, i.e., hygroscopicity, vapor permeability, and air permeability. The effect of treatment with emulsions on the comfort properties of the cotton samples is presented in Figure 10.

For each comfort index, 3 experiments were performed. Between the obtained experimental results there were no differences greater than ±1.2%. In the graphical representation of each comfort index (Figure 10), the average value resulting from the three experimental determinations was used.

The composition of the emulsions applied on the cotton support significantly influences the comfort indices. Polar groups in chitosan, gelatin, glycerin, and Tween 80 cause an increase in hygroscopicity and a decrease in vapor permeability for the treated samples compared to untreated samples. The sample treated with the E6 emulsion that did not contain wax had the highest hygroscopicity and the lowest vapor permeability. All the samples treated with emulsions showed a lower air permeability compared to the untreated sample.

#### 3.4.7. Antibacterial Activity of the Treated Textile Support

Taking into account the large number of different classes of chemical compounds existing in the composition of essential oils, it is likely that the antimicrobial activity could not be attributed to a specific mechanism but rather to a large number of target locations in the microorganism cell. The mechanism of action of the essential oils includes the degradation of the cell wall, damaging of the cytoplasmic membrane, cytoplasm coagulation, damaging of the membrane proteins, and the increase in the membrane permeability leading to leakage of the cell content. An important characteristic of the essential oil components is their hydrophobicity, which allows them to partition in the lipids of the cell membrane and mitochondria, making them more permeable; the loss of molecules and indispensable ions leads to bacterial cell death [50,66,67].

The antibacterial activity of peppermint oil was investigated against *S. aureus* and *E. coli* bacteria, which are the most frequently encountered pathogenic bacteria that affect life.

According to the Broad Institute (2010), infections due to *Escherichia coli* accounts for 17.3% of clinical infections requiring hospitalization and *Escherichia coli* represents the second most common source of infection behind *Staphylococcus aureus* (18.8%). Infections caused by these bacteria are usually treated with antibiotics but, in recent years, the emergence of some *Staphylococcus aureus* and *Escherichia coli* strains that exhibit resistance against many antibiotics, has been observed worldwide. Originating from the negative impact that antibiotics can exert on health and from the increasing resistance of some bacteria towards antibiotics, there are currently ongoing questions about the use of essential oils as agents with antibacterial properties [68,69,70,71].

Determination of the antibacterial activity for the samples treated with the stable emulsions was achieved ‘in Vitro’ by using the diffusion method in accordance with the working methodology previously described.

The antibacterial activity of the samples treated with emulsions was evaluated based on the diameter of the inhibition zone, respectively of the degree of inhibition and the IC50 Index. For the calculation of the IC50 index emulsions with a composition similar to that of the E3 emulsion were used wherein only the oil concentration and the water amounts were varied. The samples treated with the emulsions in which the oil concentrations were 2% (emulsion E3A) and 6% (emulsion E3B), denoted TE3A and TE3B, respectively, were selected to investigate the effect of essential oil concentration on antibacterial activity.

The obtained results are presented in Table 5 and Table 6 and in the Supplementary Materials (Figures S6 and S7).

The data presented show that the samples treated with the emulsions containing the peppermint essential oil exhibit an antibacterial action against both tested bacteria (*E. coli* and *S. aureus*). From the analysis of the results obtained, the following conclusions can be drawn regarding the influence of the composition of the emulsions on the antibacterial activity:

(i) The untreated cotton sample does not show antibacterial activity against the two bacteria *S. aureus* and *E. coli* used in this work;

(ii) The peppermint oil, which contains as the main components the terpene compounds menthol (62.87%), 3-p-menthene (13.16%), menthone (12.78%), and camphene (3.15%) (Figure S1 and Table S1 from the Supplementary Materials), exhibits high antimicrobial activities against Gram-positive and Gram-negative bacteria;

(iii) The diameters of the inhibition zones both for the sample treated with the emulsion TE1 (38.3 mm for *E. coli* and 53.6 mm for *S. aureus*) as well as for the sample treated with the emulsion TE3 (40.6 mm for *E. coli* and 58.3 mm for *S. aureus*), that contained beeswax, were larger compared to the diameters of the inhibition zones of the sample treated with the emulsion TE6 which did not contain wax (34.6 mm for *E. Coli* and 50.5 mm for *S. aureus*). The results obtained are in accordance with the data from the scientific literature concerning the antibacterial activity that the wax manifests against the tested bacteria [72];

(iv) The different sensitivities of the two bacterial species to the tested oil are due to the structure of their cell wall, which is different in Gram-positive bacteria compared to Gram-negative bacteria [73].

(v) Applying emulsions containing increasing amounts of oil (samples TE3A, TE3 and TE3B) to the cotton fabric increases its antibacterial activity against the tested bacteria, as can be seen from Figures S6 and S7 (Supplementary Materials), and Table 5 and Table 6. The values of the IC50 index calculated for the sample TE3 were 62.44 mg oil/g material in the case of the *E. Coli* bacteria and 56.27 mg oil/g material in the case of the *S. aureus* bacteria, respectively [74].

## 4. Conclusions

Materials with flavoring and antimicrobial properties were obtained by applying emulsions containing peppermint essential oil on cotton fabric. Several emulsion formulations containing various incorporating agents with peppermint essential oil were prepared and evaluated. The most stable emulsions were the ones that contained chitosan, gelatin, beeswax and Tween 80 (in a concentration higher than 1.50%) namely the emulsions E1, E3, and E6. Emulsions applied on cotton fabrics influence the comfort indices (increase in hygroscopicity, and a decrease in the water vapor permeability and air permeability). The amount of oil released in the artificial perspiration solution differs depending on the composition of the emulsions. For the sample TE6 (that did not contain beeswax), the release of peppermint essential oil was accomplished in a shorter time compared to the samples TE1 and TE3 (that contained beeswax). In the case where the release was performed in air, the content of volatile components that remained on the material (after 6 h) was 63.12% for the sample TE1, 74.08% for the sample TE3 and 55.65% for the sample TE6, respectively. The samples TE1 and TE3 exhibited a higher antibacterial activity against *S. aureus* and *E. coli* bacteria compared to the sample TE6. The advantages of using these emulsions to obtain materials with flavoring and antimicrobial properties consist of the following: (i) the use of essential oils extracted from mint, which is a perennial plant that multiplies very easily; (ii) gelatin and wax are relatively cheap and easy-to-procure products; (iii) the amount of synthetic emulsifier Tween 80 used for the preparation of emulsions can be lower.

From the results obtained, it can be concluded that using the application of emulsions containing peppermint essential oil on various textile supports, it is possible to obtain a varied range of patches and bandages with aromatherapeutic and antibacterial properties, that allow a controlled release of the essential oil.

## Figures and Tables

**Table 1:** Emulsion composition.

Emulsion Code	Beeswax (g)	Gelatin (g)	Chitosan 3% (g)	Glycerin (mL)	Tween 80 (mL)	M.E.O (mL)	Fresh Double Distilled Water (mL)
**E1**	4	4	12	6	2	4	68
**E2**	4	4	12	6	1	4	69
**E3**	4	4	12	6	1.5	4	68.5
**E4**	4	4	-	6	1.5	4	80.5
**E5**	4	-	12	6	1.5	4	72.5
**E6**	-	4	12	6	1.5	4	72.5

**Table 2:** pH values of the emulsions.

	Samples					
	E1	E2	E3	E4	E5	E6
**pH**	5.3	5.3	5.3	5.7	5.4	5.5

**Table 3:** Sensorial evaluation of the odor intensity for the treated samples.

Odor Intensity *			
Sample	TE1	TE3	TE6
1 day	5	5	5
2 days	4–5	4–5	4
3 days	4–5	4–5	3–4
4 days	4	4	3
5 days	3–4	3–4	2–3

* 1—very weak, 2—weak, 3—medium strong, 4—strong, 5—very strong.

**Table 4:** The obtained kinetic parameters.

Kinetic Model	Kinetic Parameters	TE1	TE3	TE6
**Korsmeyer–Peppas**	n	0.614	0.617	0.510
	k_K-P_	0.347	0.342	0.458
	R^2^	0.985	0.989	0.962

**Table 5:** Diameters of the inhibition areas for the treated samples.

Bacteria	The Diameters of the Inhibition Zones (mm) for the Samples Treated with Emulsions					
	UT (Control)	TE1	TE3	TE3A	TE3B	TE6
*E. coli*	0	38.3 ± 1.608	40.6 ± 1.705	33.3 ± 0.932	51.3 ± 1.744	34.6 ± 1.211
*S. aureus*	0	53.6 ± 2.680	58.3 ± 2.332	44.3 ± 1.683	64.0 ± 2.560	50.5 ± 1.868

**Table 6:** Inhibition degrees of the treated samples.

Bacteria	The Degree of Inhibition of the Development of Microbial Strains (%)					
	UT (Control)	TE1	TE3A	TE3	TE3B	TE6
*E. coli*	0	42.55	37.0	45.11	57.0	38.44
*S. aureus*	0	59.55	49.22	64.77	71.11	56.11

## Data Availability

Not applicable.

## Supplementary Materials

The following supporting information can be downloaded at: https://www.mdpi.com/article/10.3390/polym15102348/s1, Figure S1: GC-MS chromatogram of peppermint essential oil; Figure S2: GC-MS chromatogram of sample TE1 at 0 h; Figure S3: GC-MS chromatogram of sample TE1 at 3 h; Figure S4: GC-MS chromatogram of sample TE1 at 6 h; Figure S5: GC-MS chromatogram of sample TE3 at 0 h; Figure S6: GC-MS chromatogram of sample TE3 at 3 h; Figure S7: GC-MS chromatogram of sample TE3 at 6 h; Figure S8: GC-MS chromatogram of sample TE6 at 0 h; Figure S9: GC-MS chromatogram of sample TE6 at 3 h; Figure S10: GC-MS chromatogram of sample TE6 at 6 h; Figure S11: Antimicrobial activity of the untreated sample (a) and of the treated samples TE1 (b), TE6 (c), TE3 (d), TE3A (e) and TE3B (f) against E. coli.; Figure S12: Antimicrobial activity of the untreated sample (a) and of the treated samples TE1 (b), TE6 (c), TE3 (d), TE3A (e) and TE3B (f) against S.aureus; Table S1: Chemical composition of peppermint essential oil; Table S2: Identified compounds, peak area and area percentage in sample TE1.0 at 0 h; Table S3: Identified compounds, peak area and area percentage in sample TE1.3 at 3 h; Table S4: Identified compounds, peak area and area percentage in sample TE1.6 at 6 h; Table S5: Identified compounds, peak area and area percentage in sample TE3.0 at 0 h; Table S6: Identified compounds, peak area and area percentage in sample TE3.3 at 3 h; Table S7: Identified compounds, peak area and area percentage in sample TE3.6 at 6 h; Table S8: Identified compounds, peak area and area percentage in sample TE6.0 at 0 h; Table S9: Identified compounds, peak area and area percentage in sample TE6.3 at 3 h; Table S10: Identified compounds, peak area and area percentage in sample TE6.6 at 6 h; Table S11: Total peak area of volatile compounds and Area% for sample TE1; Table S12: Total peak area of volatile compounds and Area% for sample TE3; Table S13: Total peak area of volatile compounds and Area% for sample TE6.
