# Supplementary material for: Aromatherapeutic and Antibacterial Properties of Cotton Materials Treated with Emulsions Containing Peppermint Essential Oil (Menthae piperitae aetheroleum)
Source: Polymers (Basel). 2023 May 17;15(10):2348. doi: 10.3390/polym15102348 (PMC10221490; doi:10.3390/polym15102348)
Supplement: Supplementary file 1 [file polymers-15-02348-s001.zip › polymers-2355712-supplementary.pdf]

polymers

Supplementary Materials

Identified Compounds, Peak Area and Area Percentage

The data obtained after the analysis of the samples are presented in Figures S1–12 and Tables S1–13

1. GC-MS Chromatograms

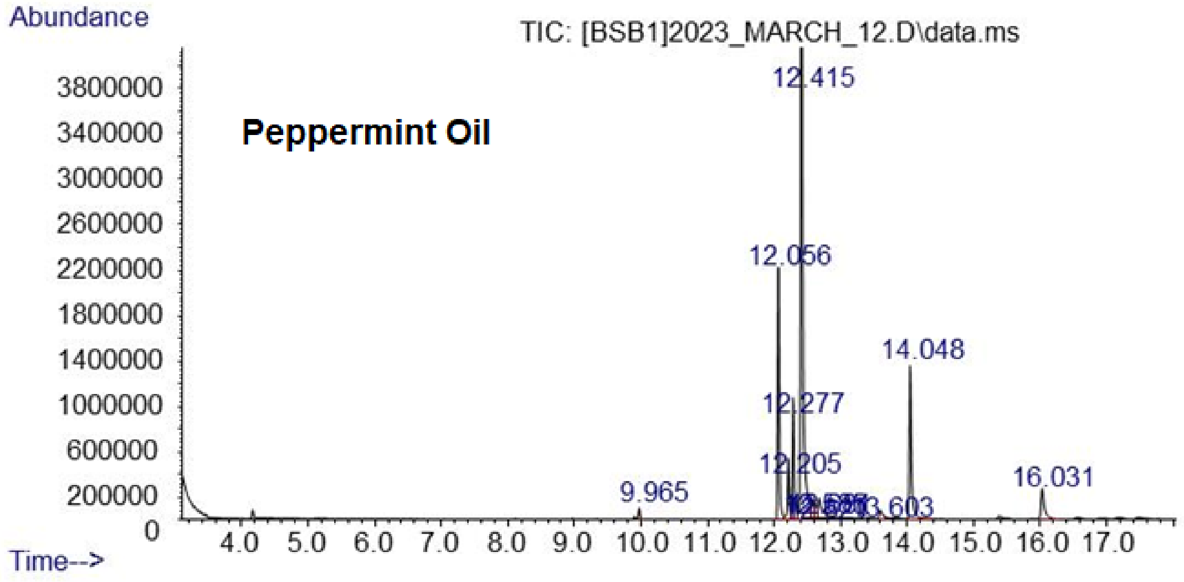

Figure S1. GC-MS chromatogram of peppermint essential oil.

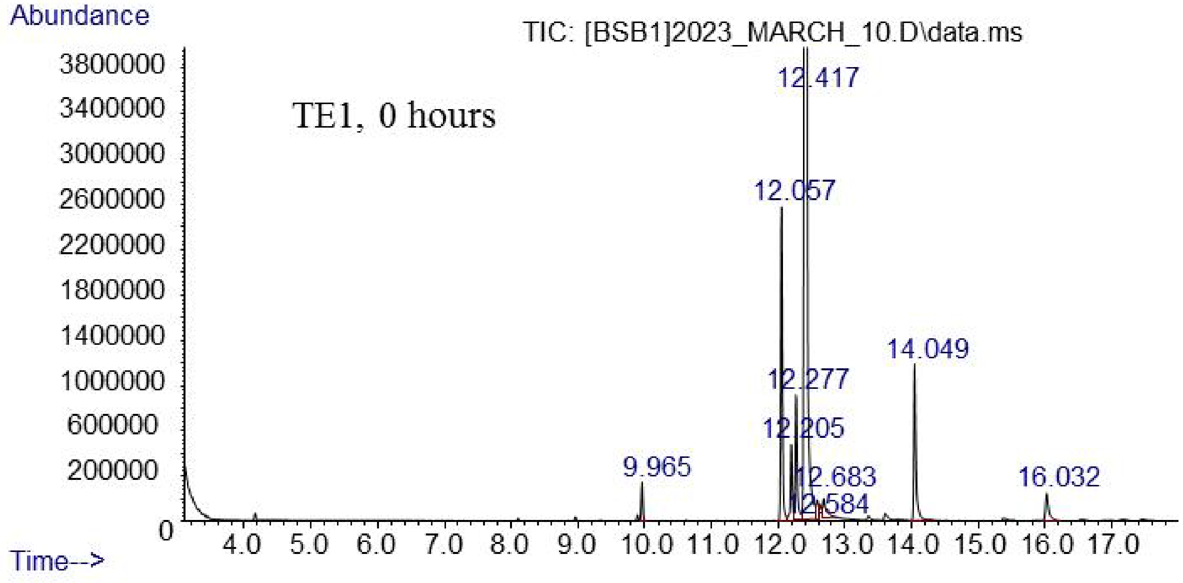

Figure S2. GC-MS chromatogram of sample TE1 at 0 hours.

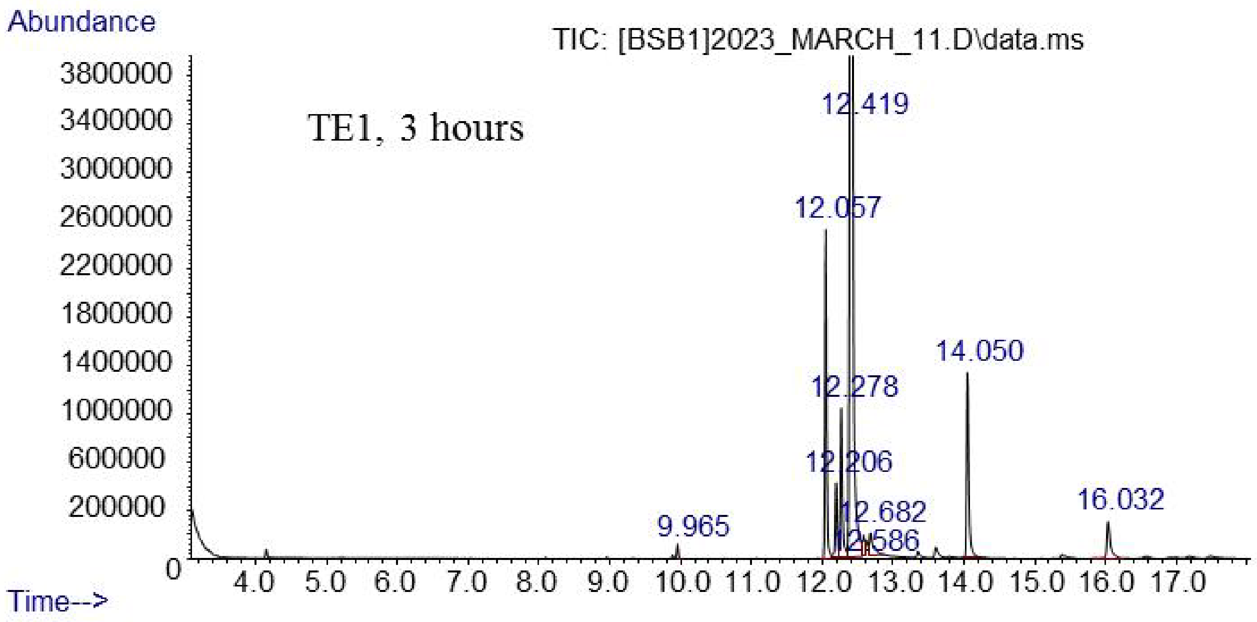

Figure S3. GC-MS chromatogram of sample TE1 at 3 hours.

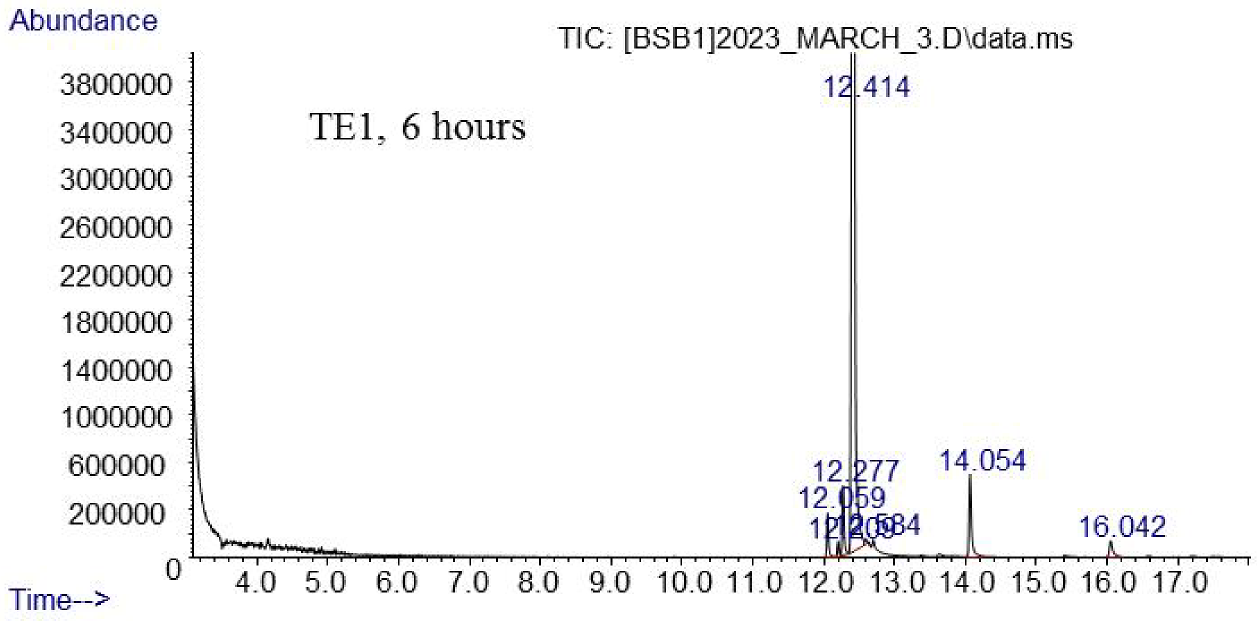

Figure S4. GC-MS chromatogram of sample TE1 at 6 hours.

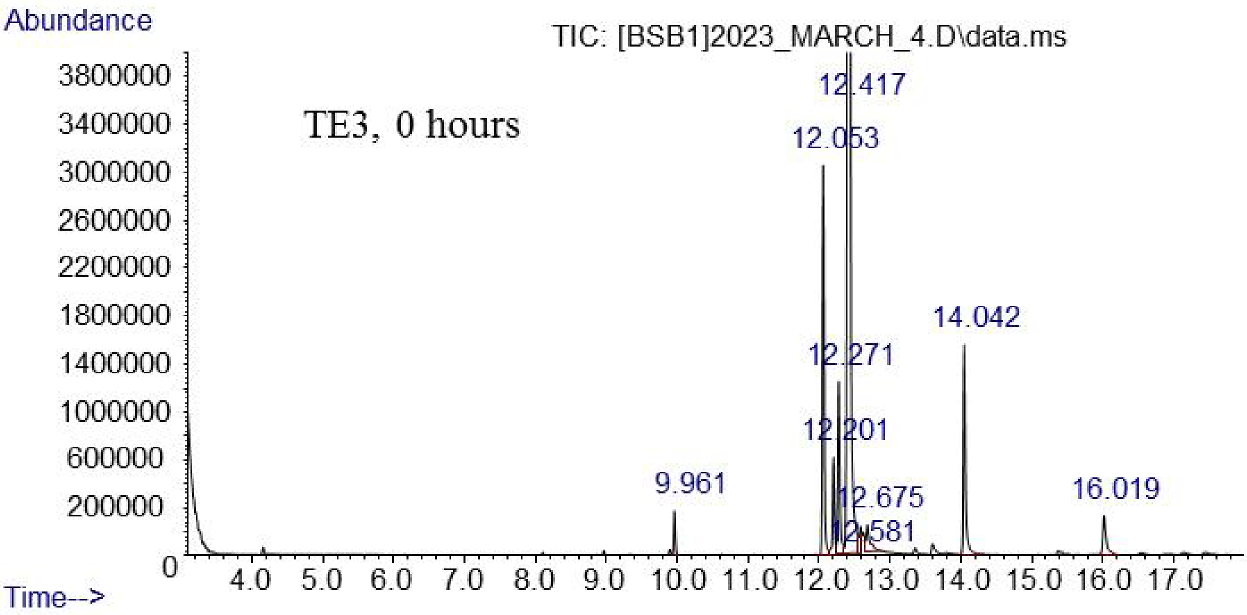

Figure S5. GC-MS chromatogram of sample TE3 at 0 hours.

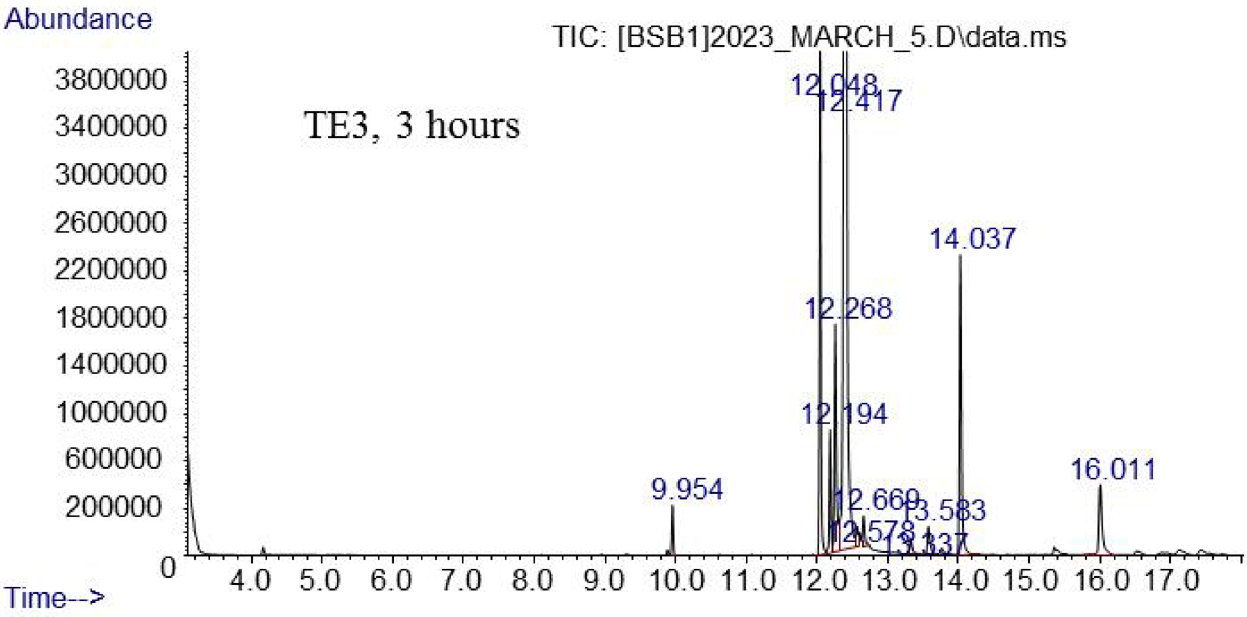

Figure S6. GC-MS chromatogram of sample TE3 at 3 hours.

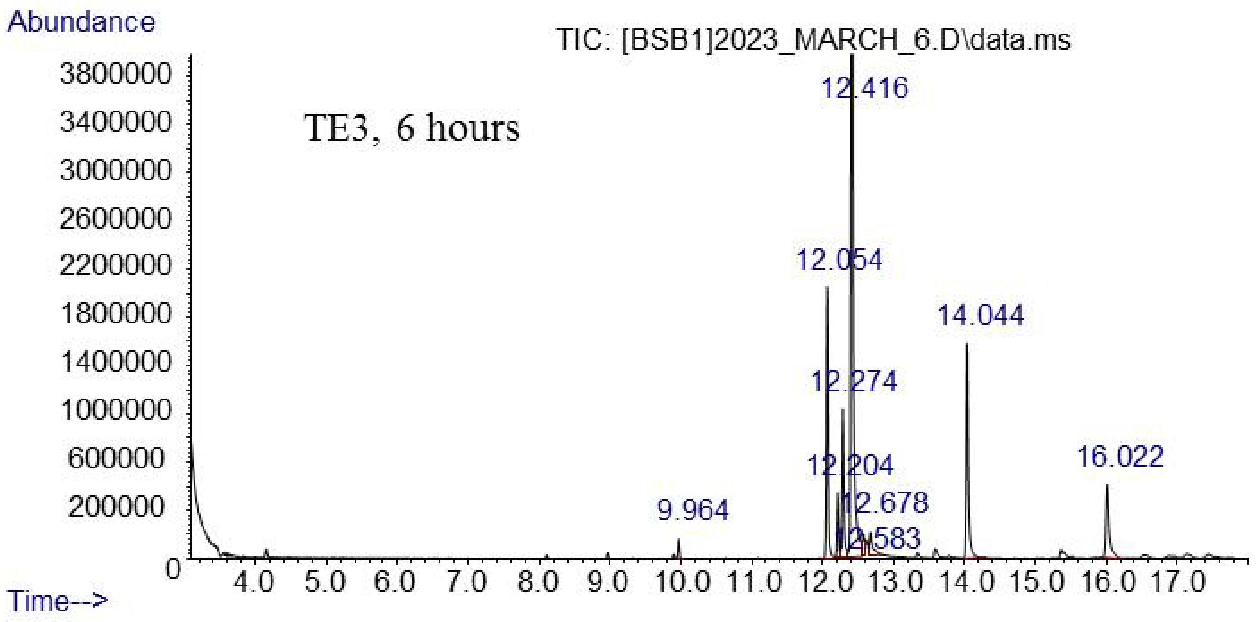

Figure S7. GC-MS chromatogram of sample TE3 at 6 hours.

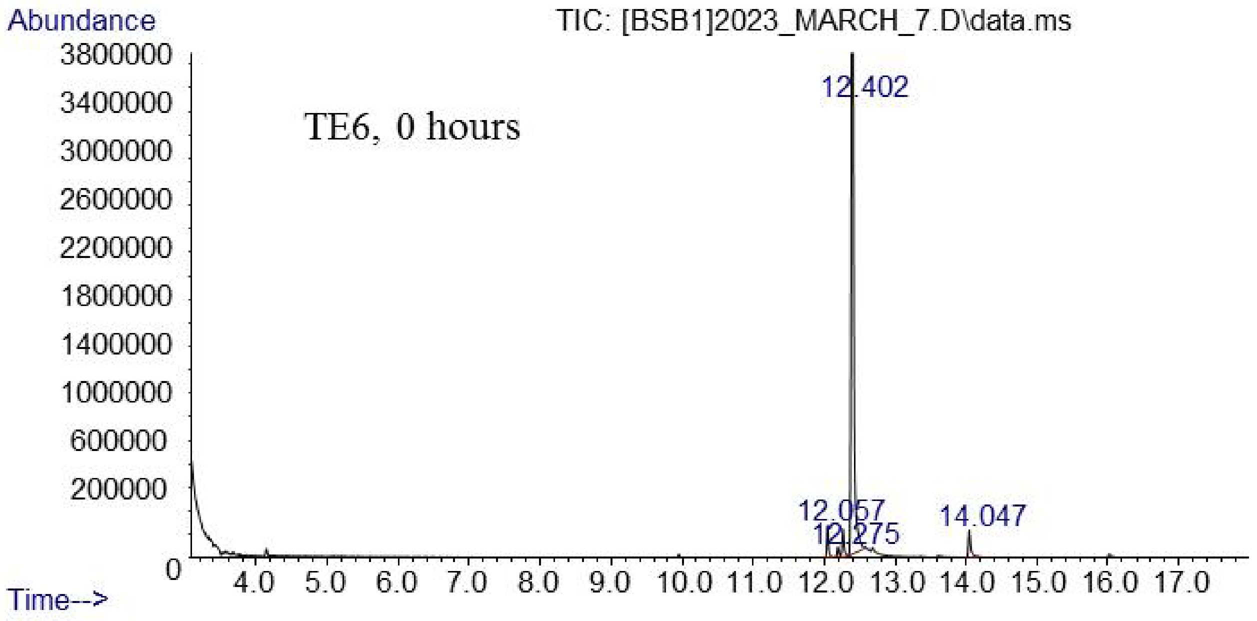

Figure S8. GC-MS chromatogram of sample TE6 at 0 hours.

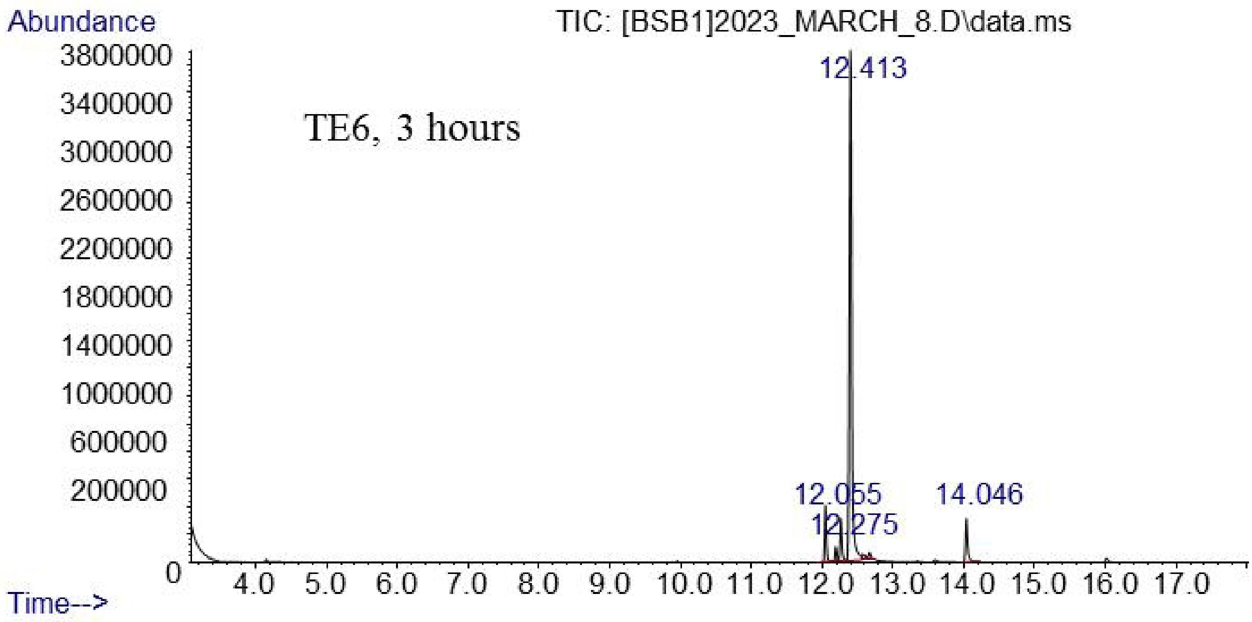

Figure S9. GC-MS chromatogram of sample TE6 at 3 hours.

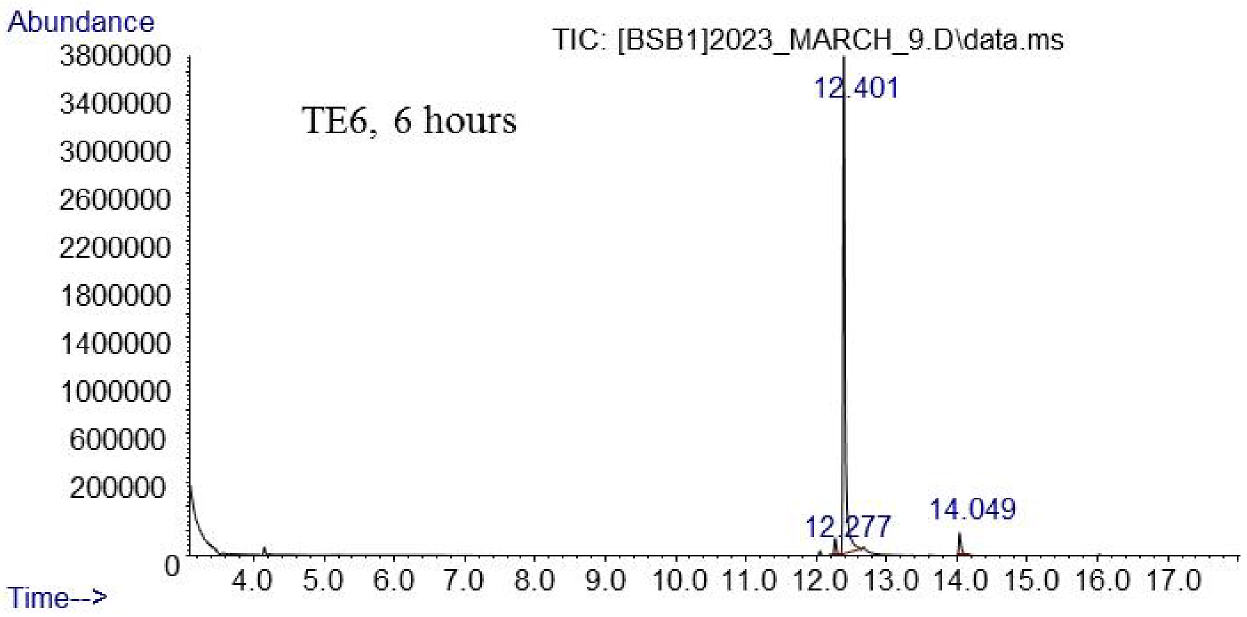

Figure S10. GC-MS chromatogram of sample TE6 at 6 hours.

2. Antimicrobial Activity

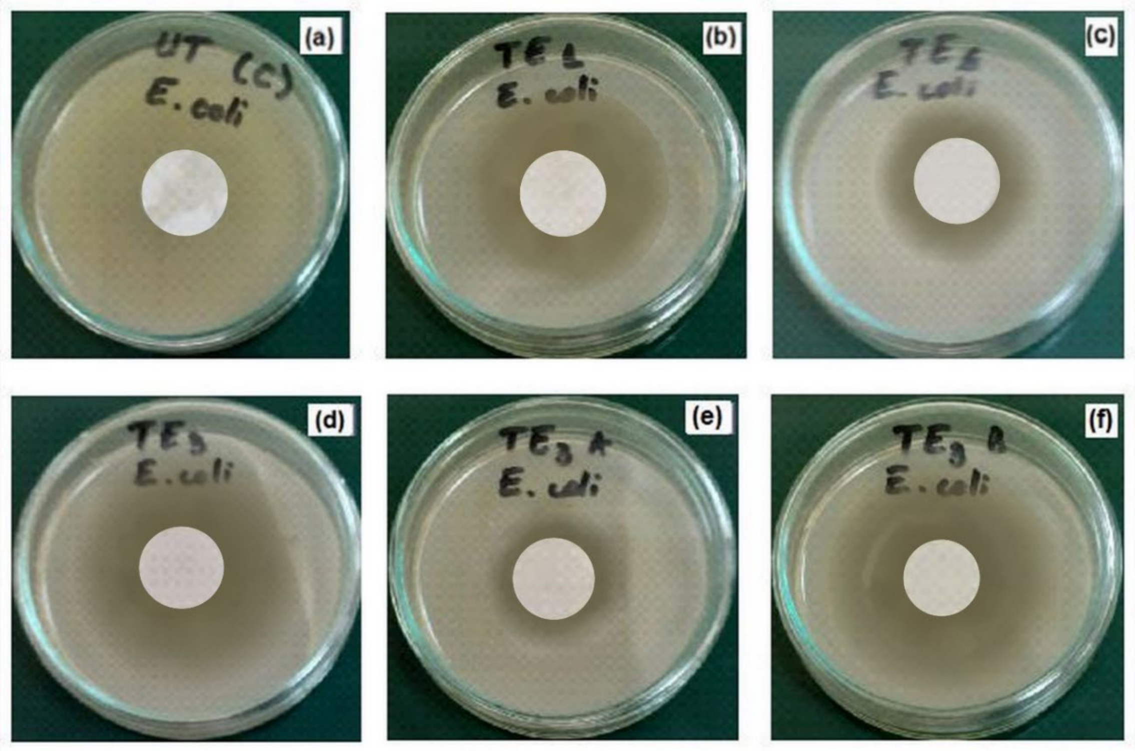

Figure S11. Antimicrobial activity of the untreated sample (a) and of the treated samples TE1 (b), TE6 (c), TE3 (d), TE3A (e) and TE3B (f) against *E. coli.*

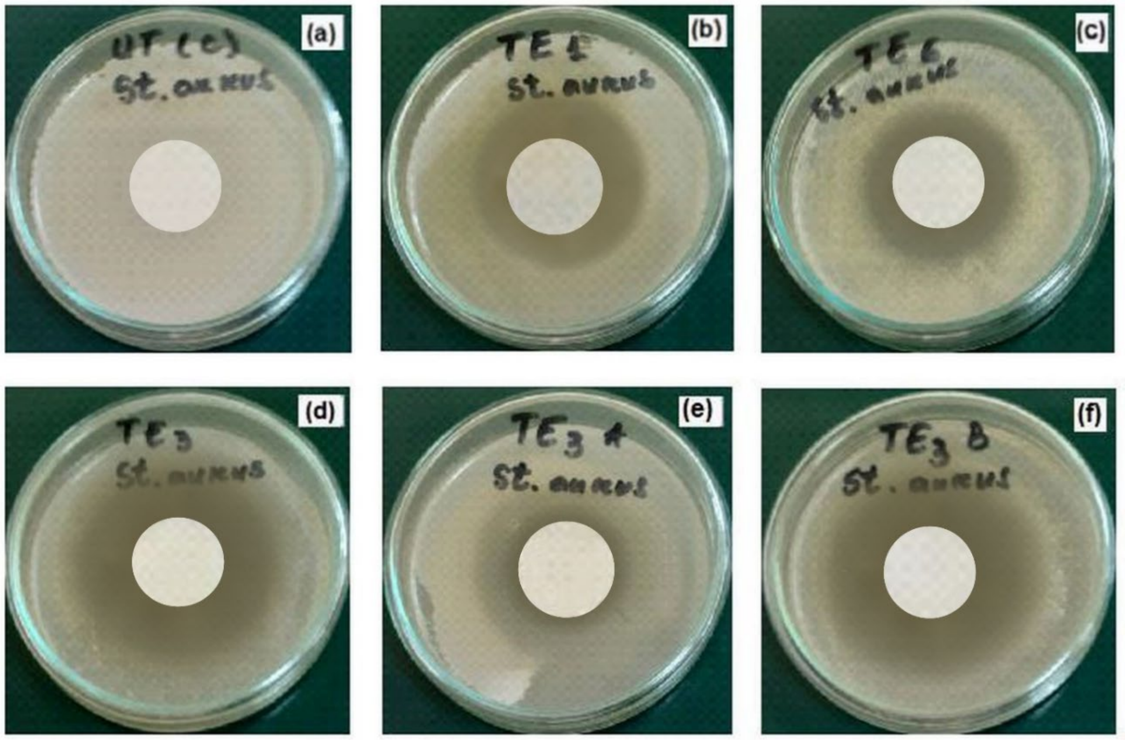

Figure S12. Antimicrobial activity of the untreated sample (a) and of the treated samples TE1 (b), TE6 (c), TE3 (d), TE3A (e) and TE3B (f) against *S.aureus.*

3. Chemical Composition Determined Using the GCSM Method

Table S1. Chemical composition of peppermint essential oil.

Pk#	Rt	Name (CAS)	Name	Area %
1	8.103	2,5-Cyclohexadiene-1-carboxylic acid, 1-methyl-, methyl ester (CAS)		0.03
2	8.964	7-Methyl-3-methylene-1,6-octadiene	Myrcene	0.05
3	9.897	Cyclohexene, 1-methyl-4-(1-methylethenyl)-, (S)-	Limonene	0.12
4	9.962	2-Oxabicyclo[2.2.2]octane, 1,3,3-trimethyl- (CAS)	1,8-Cineole	0.42
5	12.056	Cyclohexanone, 5-methyl-2-(1-methylethyl)- (CAS)	Menthone	10.12
6	12.206	Cyclohexanone, 5-methyl-2-(1-methylethyl)- (CAS)	Menthone	2.66
7	12.278	Cyclohexene, 4-methyl-1-(1-methylethyl)- (CAS)	3-p-Menthene	5.67
8	12.415	Cyclohexanol, 5-methyl-2-(1-methylethyl)-, (1.alpha.,2.beta.,5.alpha.)-(.+-.)-	Menthol, (.+-.)-	62.87
9	12.584	Bicyclo[4.1.0]heptane, 3,7,7-trimethyl-, (1.alpha.,3.alpha.,6.alpha.)- (CAS)	trans-Carane	1.93
10	12.624			
11	12.682	Bicyclo[2.2.1]heptane, 2,2-dimethyl-3-methylene- (CAS)	Camphene	3.15
12	13.354	Cyclohexanone, 5-methyl-2-(1-methylethylidene)-, (R)- (CAS)	Pulegone	0.5
13	13.602	2-Cyclohexen-1-one, 3-methyl-6-(1-methylethyl)- (CAS)	Piperitone	0.79
14	13.791	3,5-Octadiene, 2,7-dimethyl-, (E,Z)- (CAS)		0.22
15	14.046	Cyclohexene, 4-methyl-1-(1-methylethyl)- (CAS)	3-p-Menthene	7.49
16	15.383	Cyclobuta[1,2:3,4]dicyclopentene, decahydro-3a-methyl-6-methylene-1-(1-methylethyl)-, [1S-(1.alpha.,3a.alpha.,3b.beta.,6a.beta.,6b.alpha.)]- (CAS)	.beta.-Bourbonene	0.13
17	15.422	Bicyclo[3.2.0]hept-3-en-6-one, 4,7,7-trimethyl-	Filifolone	0.09
18	16.029	Bicyclo[7.2.0]undec-4-ene, 4,11,11-trimethyl-8-methylene-, [1R-(1R*,4E,9S*)]- (CAS)	.beta.-Caryophyllene	2.59
19	16.55	1,4,8-CycloundE,E,E)- (CAS)	.alpha.-Humulene	0.28
20	16.903	1-Methyl-5-methylene-8-isopropyl-1,6-cyclodecadiene	Germacrene-D	0.12
21	16.974	1H-Cycloprop[e]azulene, decahydro-1,1,7-trimethyl-4-methylene-, [1aR-(1a.alpha.,4a.beta.,7.alpha.,7a.beta.,7b.alpha.)]- (CAS)	Alloaromadendrene	0.05
22	17.17	Naphthalene, 1,2,4a,5,6,8a-hexahydro-4,7-dimethyl-1-(1-methylethyl)-, (1.alpha.,4a.alpha.,8a.alpha.)- (CAS)	.alpha.-Muurolene	0.33
23	17.483	Tricyclo[4.4.0.0(2,7)]dec-3-ene, 1,3-dimethyl-8-(1-methylethyl)-, stereoisomer (CAS)	.alpha.-Copaene	0.38

Table S2. Identified compounds, peak area and area percentage in sample TE1.0 at 0 hours.

Pk#	Rt	Name (CAS)	Name	Peak Area	Area %
1	9.897	Cyclohexene, 1-methyl-4-(1-methylethenyl)-, (S)- (CAS)	l-Limonene	928871	0.23
2	9.962	2-Oxabicyclo[2.2.2]octane, 1,3,3-trimethyl- (CAS)	1,8-Cineole	5466465	1.38
3	12.056	Cyclohexanone, 5-methyl-2-(1-methylethyl)- (CAS)	Menthone	60454279	15.25
4	12.206				
5	12.278	Cyclohexane, 1-methyl-4-(1-methylethylidene)- (CAS)	4(8)-p-Menthene	22921087	5.78
6	12.415	Cyclohexanol, 5-methyl-2-(1-methylethyl)-, [1R-(1.alpha.,2.beta.,5.alpha.)]- (CAS)	Menthol	248126474	62.6
7	12.584	Bicyclo[4.1.0]heptane, 3,7,7-trimethyl- (CAS)	Carane	7589047	1.92
8	12.624				
9	12.682	Bicyclo[2.2.1]heptane, 2,2-dimethyl-3-methylene- (CAS)	Camphene	9175667	2.31
10	13.609		Piperitone	2233647	0.56
11	14.046	Cyclohexene, 4-methyl-1-(1-methyle	3-p-Menthene (Menthomenthene)	30744624	7.76
12	16.035	Bicyclo[7.2.0]undec-4-ene, 4,11,11-trimethyl-8-methylene-, [1R-(1R*,4E,9S*)]- (CAS)	.BETA.-Caryophyllene	8747829	2.21
			Total	396387990	

Table S3. Identified compounds, peak area and area percentage in sample TE1.3 at 3 hours.

Pk#	Rt	Name (CAS)	Name	Peak Area	Area %
1	9.962	2-Oxabicyclo[2.2.2]octane, 1,3,3-trimethyl- (CAS)	1,8-Cineole	1766896	0.48
2	12.056	Cyclohexanone, 5-methyl-2-(1-methylethyl)- (CAS)	Menthone	51707145	14.03
3	12.206				
4	12.278	Cyclohexane, 1-methyl-4-(1-methylethylidene)- (CAS)	4(8)-p-Menthene	21847967	5.93
5	12.421	Cyclohexanol, 5-methyl-2-(1-methylethyl)-, [1R-(1.alpha.,2.beta.,5.alpha.)]- (CAS)	Menthol	240972890	65.39
6	12.584	Bicyclo[4.1.0]heptane, 3,7,7-trimethyl-, (1.alpha.,3.alpha.,6.alpha.)- (CAS)	trans-Carane	4197210	1.14
7	12.623				
8	12.682	Bicyclo[2.2.1]heptane, 2,2-dimethyl-3-methylene- (CAS)	Camphene	2813396	0.76
9	13.354	Pulegone	Pulegone	1404062	0.38
10	13.602	Piperitone	Piperitone	2848759	0.77
11	14.052	Cyclohexene, 4-methyl-1-(1-methylethyl)- (CAS)	3-p-Menthene	30858819	8.37
12	16.035	.BETA.-Caryophyllene	.beta.-Caryophyllene	10104471	2.74
			Total	368521615	

Table S4. Identified compounds, peak area and area percentage in sample TE1.6 at 6 hours.

Pk#	Rt	Name (CAS)	Name	Peak Area	Area%
1	12.056	Cyclohexanone, 5-methyl-2-(1-methylethyl)- (CAS)	Menthone	8960467	3.58
2	12.213				
3	12.278	Cyclohexane, 1-methyl-4-(1-methylethylidene)- (CAS)	p-Menth-4(8)-ene	12444760	4.97
4	12.415	Cyclohexanol, 5-methyl-2-(1-methylethyl)-, [1R-(1.alpha.,2.beta.,5.alpha.)]- (CAS)	L-(-)-Menthol	192997105	77.14
5	12.584	Bicyclo[4.1.0]heptane, 3,7,7-trimethyl- (CAS)	Carane	6658263	2.66
6	12.624				
7	12.682	Bicyclo[2.2.1]heptane, 2,2-dimethyl-3-methylene- (CAS)	Camphene	7749386	3.1
8	14.052	Cyclohexene, 4-methyl-1-(1-methylethyl)- (CAS)	Menthomenthene	16360436	6.54
9	16.042	Bicyclo[7.2.0]undec-4-ene, 4,11,11-trimethyl-8-methylene-, [1R-(1R*,4E,9S*)]- (CAS)	.beta.-Caryophyllene	5010578	2
			Total	250180995	99.99

Table S5. Identified compounds, peak area and area percentage in sample TE3.0 at 0 hours.

Pk#	Rt	Name (CAS)	Name	Peak Area	Area %
1	9.962	2-Oxabicyclo[2.2.2]octane, 1,3,3-trimethyl- (CAS)	1,8-Cineole	6905461	1.26
32	12.056	Cyclohexanone, 5-methyl-2-(1-methylethyl)- (CAS)	Menthone	82324153	1.50
3	12.2				
4	12.271	Cyclohexene, 4-methyl-1-(1-methylethyl)- (CAS)	3-p-Menthene	34758370	6.33
5	12.415	Cyclohexanol, 5-methyl-2-(1-methylethyl)-, (1.alpha.,2.beta.,5.alpha.)-(.+-.)-	Menthol, (.+-.)-	343338314	62.57
6	12.578	Bicyclo[4.1.0]heptane, 3,7,7-trimethyl- (CAS)	Carane	10221319	1.86
7	12.617				
8	12.676	Bicyclo[2.2.1]heptane, 2,2-dimethyl-3-methylene- (CAS)	CAMPHENE	11147153	2.03
9	13.596	2-Cyclohexen-1-one, 3-methyl-6-(1-methylethyl)- (CAS)	Piperitone	3533417	0.64
10	14.039	Cyclohexene, 4-methyl-1-(1-methylethyl)- (CAS)	3-p-Menthene	43285553	7.89
11	16.016	Cyclohexanol, 5-methyl-2-(1-methylethyl)-, (1.alpha.,2.beta.,5.alpha.)-(.+-.)-	Menthol, (.+-.)-	13212265	2.41
			Total	548726005	100

Table S6. Identified compounds, peak area and area percentage in sample TE3.3 at 3 hours.

Pk#	Rt	Name (CAS)	Name	Peak Area	Area %
1	9.956	2-Oxabicyclo[2.2.2]octane, 1,3,3-trimethyl- (CAS)	1,8-Cineole	5987628	1.20
2	12.049	Cyclohexanone, 5-methyl-2-(1-methylethyl)- (CAS)	Menthone	83691924	16.81
3	12.193				
4	12.265	Cyclohexene, 4-methyl-1-(1-methylethyl)- (CAS)	3-p-Menthene	78330671	15.74
11	14.039				
5	12.415	Cyclohexanol, 5-methyl-2-(1-methylethyl)-, [1R-(1.alpha.,2.beta.,5.alpha.)]- (CAS)	1-Menthol	295462908	59.35
6	12.578	Bicyclo[4.1.0]heptane, 3,7,7-trimethyl-(CAS)	Carane	5055044	1.02
7	12.61				
8	12.669	Bicyclo[2.2.1]heptane, 2,2-dimethyl-3-methylene- (CAS)	Camphene	4293082	0.86
9	13.335	Cyclohexanone, 5-methyl-2-(1-methylethylidene)-, (R)- (CAS)	Pulegone	2804228	0.56
10	13.582	2-Cyclohexen-1-one, 3-methyl-6-(1-methylethyl)- (CAS)	Piperitone	5209313	1.05
12	15.363	Cyclobuta[1,2:3,4]dicyclopentene, decahydro-3a-methyl-6-methylene-1-(1-methylethyl)-, [1S-(1.alpha.,3a.alpha.,3b.beta.,6a.beta.,6b.alpha.)]- (CAS)	.beta.-Bourbonene	350411	0.07
13	16.009	Bicyclo[7.2.0]undec-4-ene, 4,11,11-trimethyl-8-methylene-, [1R-(1R*,4E,9S*)]- (CAS)	.beta.-Caryophyllene	16618124	3.34
			Total	497803334	100

Table S7. Identified compounds, peak area and area percentage in sample TE3.6 at 6 hours.

Pk#	Rt	Name (CAS)	Name	Peak Area	Area %
1	9.962	2-Oxabicyclo[2.2.2]octane, 1,3,3-trimethyl- (CAS)	1,8-Cineole	2526635	0.62
2	12.056	Cyclohexanone, 5-methyl-2-(1-methylethyl)- (CAS)	Menthone	47949030	11.79
3	12.206				
4	12.271	Cyclohexane, 1-methyl-4-(1-methylethylidene)- (CAS)	4(8)-p-Menthene	24203987	5.95
5	12.415	Cyclohexanol, 5-methyl-2-(1-methylethyl)-, (1.alpha.,2.beta.,5.alpha.)-(.+-.)-	Menthol, (.+-.)-	255014388	62.74
6	12.584	Bicyclo[4.1.0]heptane, 3,7,7-trimethyl- (CAS)	Carane	7862684	1.93
7	12.617				
8	12.676	Bicyclo[2.2.1]heptane, 2,2-dimethyl-3-methylene- (CAS)	Camphene	8854971	2.18
9	13.595	2-Cyclohexen-1-one, 3-methyl-6-(1-methylethyl)- (CAS)	Piperitone	2468819	0.61
10	14.045	Cyclohexene, 4-methyl-1-(1-methylethyl)- (CAS)	3-p-Menthene	37228398	9.16
11	15.376	Cyclobuta[1,2:3,4]dicyclopentene, decahydro-3a-methyl-6-methylene-1-(1-methylethyl)-, [1S-(1.alpha.,3a.alpha.,3b.beta.,6a.beta.,6b.alpha.)]-(CAS)	.beta.-Bourbonene	261444	0.06
12	16.022	Bicyclo[7.2.0]undec-4-ene, 4,11,11-trimethyl-8-methylene-, [1R-(1R*,4E,9S*)]- (CAS)	.beta.-Caryophyllene	20105039	4.95
			Total	406475395	99.99

Table S8. Identified compounds, peak area and area percentage in sample TE6.0 at 0 hours.

Pk#	Rt	Name (CAS)	Name	Peak Area	Area %
1	9.962	2-Oxabicyclo[2.2.2]octane, 1,3,3-trimethyl- (CAS)	1,8-Cineole	659285	0.32
2	12.056	Cyclohexanone, 5-methyl-2-(1-methylethyl)- (CAS)	Menthone	9969287	4.9
3	12.206				
4	12.278	Cyclohexane, 1-methyl-4-(1-methylethylidene)- (CAS)	4(8)-p-Menthene	7808433	3.84
5	12.402	Cyclohexanol, 5-methyl-2-(1-methylethyl)-, (1.alpha.,2.beta.,5.alpha.)-(.+-.)-	Menthol, (.+-.)-	162201717	79.72
6	12.578	Bicyclo[4.1.0]heptane, 3,7,7-trimethyl-, (1.alpha.,3.alpha.,6.alpha.)- (CAS)	trans-Carane	3129590	1.54
7	12.617	Bicyclo[4.1.0]heptane, 3,7,7-trimethyl- (CAS)	Carane	2590853	1.27
8	12.682	Bicyclo[4.1.0]hept-3-ene, 3,7,7-trimethyl- (CAS)	.delta.3-Carene	6824229	3.35
9	14.046	Cyclohexene, 4-methyl-1-(1-methylethyl)- (CAS)	3-p-Menthene	8616714	4.24
10	16.022	Bicyclo[7.2.0]undec-4-ene, 4,11,11-trimethyl-8-methylene-, [1R-(1R*,4E,9S*)]- (CAS)	.beta.-Caryophyllene	1659216	0.82
			Total	203459322	100

Table S9. Identified compounds, peak area and area percentage in sample TE6.3 at 3 hours.

Pk#	Rt	Name (CAS)	Name	Peak Area	Area %
1	9.962	2-Oxabicyclo[2.2.2]octane, 1,3,3-trimethyl- (CAS)	1,8-Cineole	395415	0.22
2	12.056	Cyclohexanone, 5-methyl-2-(1-methylethyl)- (CAS)	Menthone	14468297	7.89
3	12.206				
4	12.278	Cyclohexene, 4-methyl-1-(1-methylethyl)- (CAS)	3-p-Menthene	21181642	11.55
10	14.046				
5	12.415	Cyclohexanol, 5-methyl-2-(1-methylethyl)-, (1.alpha.,2.beta.,5.alpha.)-(.+-.)-	Menthol, (.+-.)-	140003637	76.37
6	12.584	Bicyclo[4.1.0]heptane, 3,7,7-trimethyl- (CAS)	Carane	2564121	1.4
7	12.617				
8	12.682	Bicyclo[4.1.0]hept-3-ene, 3,7,7-trimethyl- (CAS)	.delta.3-Carene	1726149	0.94
9	13.602	2-Cyclohexen-1-one, 3-methyl-6-(1-methylethyl)- (CAS)	Piperitone	1131120	0.62
11	16.029	Bicyclo[7.2.0]undec-4-ene, 4,11,11-trimethyl-8-methylene-, [1R-(1R*,4E,9S*)]- (CAS)	trans-Caryophyllene	1852343	1.01
			Total	183322723	100

Table S10. Identified compounds, peak area and area percentage in sample TE6.6 at 6 hours.

Pk#	Rt	Name (CAS)	Name	Peak Area	Area %
1	12.063	Cyclohexanone, 5-methyl-2-(1-methylethyl)-, cis- (CAS)	ISO MENTHONE	1031200	0.91
2	12.206				
3	12.278	Cyclohexene, 4-methyl-1-(1-methylethyl)- (CAS)	3-p-Menthene	8191966	7.23
9	14.052				
4	12.402	Cyclohexanol, 5-methyl-2-(1-methylethyl)-, (1.alpha.,2.beta.,5.alpha.)-(.+-.)-	Menthol, (.+-.)-	93985485	83
5	12.578	Bicyclo[4.1.0]heptane, 3,7,7-trimethyl- (CAS)	Carane	3540972	3.12
6	12.617				
7	12.689	Bicyclo[2.2.1]heptane, 2,2-dimethyl-3-methylene- (CAS)	Camphene	5911683	5.22
8	13.615	1,3-Dimethyl-1-cyclohexene		163955	0.14
10	16.029	EXO-BICYCLO[4.1.0]HEPT-3-EN-7-CARBAMIC ACID		408734	0.36
			Total	113233995	99.98

Table S11. Total peak area of volatile compounds and Area% for sample TE1.

Sample code	Sum of the peak areas	Sum of the peak areas (%)
TE1.0	396387990	100.00
TE1.3	368521615	92.97
TE1.6	250180995	63.12

Table S12. Total peak area of volatile compounds and Area% for sample TE3.

Sample code	Sum of the peak areas	Sum of the peak areas (%)
TE3.0	548726005	100.00
TE3.3	497803334	90.72
TE3.6	406475395	74.08

Table S13. Total peak area of volatile compounds and Area% for sample TE6.

Sample code	Sum of the peak areas	Sum of the peak areas (%)
TE6.0	203459322	100
TE6.3	183322723	90.10
TE6.6	113233995	55.65
